# Introductory Lectures

**Published:** 1849

**Authors:** 


### ARTICLE V.

*Introductory Lectures, for the Sessions of* 1848–9,

By James Blake, M. D., Prof., &c., St. Louis University.
" Geo. R. Grant, Med. Prof., &c., Memphis Med. College.
" By Samuel Henry Dickson, M. D., Prof., &c., New York University.
" John Wiltbank, M.D., Prof., &c., Pennsylvania Col.
" Frederick Merrick, A. M., M. D., Prof., &c., Starling Med. College.
" E. S. Cooper, M. D., Peoria Medical Courses. (private.)

Prof. Blake's introductory, the first of these, is an account of some of the encouragements that attend the prosecution of the study of medical science, with clear reasoning upon their tendencies, and some advice in reference to the best plan of study to avoid fallacies, both in regard to the observance of facts and drawing conclusions from them.

Prof. Grant's, after arguing the importance of starting right, and of system in efforts at study, gives short biographical sketches of some of the most eminent men of our profession in former times, such as Harvey, Sydenham, Boorheave, Hoffman, William and John Hunter, Cullen, Jenner, Rush, Physick, and Depuytren, with a view of showing his class the importance of constant and continued application, and to hold out inducements to them to aim high and devote themselves to the profession.

Prof. Dickson's is upon the subject of Hygiene, which is treated under the following heads: 1st. The physical education of the young. 2d. Personal Hygiene—the course of life of the individual. 3. Public or municipal hygiene, not inaptly treated of by a recent writer as "the political economy of health."

It contains a large collection of interesting facts on the subject of hygiene, and many important practical suggestions in reference to education of children, influence of climate, personal cleanliness, quarantine, and public regulations in reference to density of population in our large cities.

The first of these points, the impropriety of urging children forward in walking or mental exercise in early infancy, is correctly dwelt upon. And when children are sent to school, the following rules are advised:

I would regulate the hour of study in a general ratio to the age of the child; between three and five years of age, three hours a day of school discipline are as much as can be allowed; from five to ten, we may impose five hours daily of study and confinement, but no more; from ten to fourteen, six or seven hours may be spent in preparing and reciting lessons, and in undergoing all instruction and practice in whatever departments. Sir Thomas More, in his exquisitely imagined Utopia, does not allow more than seven hours of regular labor to be allotted to any one.

I fully agree with the venerable author above quoted in questioning the propriety of "the application of the system of rivalry," as he phrases it, "to the softer sex;" of arousing in them the spirit of emulation—the ambition to excel. He speaks charmingly of the success of principles and motives of higher character and better adapted to these more pliant subjects —the force of reason, the sense of duty, the desire to be loved, and the patient and kindly influence of the good teacher.

If I admitted of the distribution of premiums at all among girls, it should be for gentleness, docility, goodness; but for no form of cleverness. Among boys there is no substitute for the great motive of the manly breast—ambition; but it must not be too strongly stimulated. Applying it cautiously, I would always aid it with the most familiar impelling power of the older world, a favorite clearly of the wise Solomon—the time honored rod, which it is too much the fashion of the present day, and in the western hemisphere especially, to neglect.

Upon the second point, after speaking in glowing terms of the conquests of our race in overcoming the barriers to its progress found in the burning rays of the torrid zone, and the extreme coldness of the polar regions, he says:

But I would abandon, after due exertion, every unreasonable and hopeless enterprize. I would leave to the savage tribes, fitted by the very inferiority of their attribntes to roam over the wilds of the great American deserts of Oregon, of New Mexico, and of California, these desolate domains; I would cease to contest with the Bedouin for his torrid sands, and to the African I would abandon the unmolested enjoyment of his thick mangrove jungle, and steaming morass. The sacrifice of life and health in the Eastern Colonies of the British, in their attempts to fix themselves upon the Coast of Guinea, and the Islands of the Western Archipelago, and in their commercial explorations of the Niger and Tsadda, offer abundant warning of the absolute impossibility of success in similar projects, and show the insurmountable opposition of climatic influences. Yet the energy of our Anglo-Norman character is so irrepressible that I should feel no surprise at learning that an expedition was on foot to make a settlement upon the icy promontories of Boothia Felix, or invade the Abyssinian mountains. It is our "manifest destiny" to roll our restless waves of burning life against every barrier, and to dash ourselves into foam against every obstacle over which we cannot sweep in triumph and success.

It is certain that in reference to the American localities here specified, we have no marks of insalubrity to deter us from carrying the conquests of civilization over them and of planting there colonies—yes, great and populous states—of Anglo-Norman inhabitants. On the contrary, from the tide of emigration now set in the direction of those *desert wilds*, we fancy that should this proposition to abandon them to the savage tribes, whose inferiority fits them for the country, fall into the hands of inhabitants of those countries or even of the author himself five years hence, they would have great difficulty in admitting that such sentiments could have been advanced so late as the winter of 1848-9. In fact, we have serious doubts if there be any country for the occupancy of which superiority disqualifies man; on the contrary, knowledge of science and art enables him to protect and take care of himself so as to profitably occupy situations otherwise uninhabitable to man. True, a change of climate is frequently fatal; but where is the spot upon which the Caucasian cannot as well as any other variety of the human family—yes, better—live and thrive, after acclimation or where reared on the spot?

After speaking of the causes of the greater prevalence of insanity in the northern states of America than elsewhere, which he correctly attri butes to constant and intense application, the Sabbath, that divine hygienic regulation for the relief of wearied nature, is described in beautiful colors.

He thinks we as a nation bathe less than the inhabitants of most other civilized countries. The following are pertinent remarks:

I attribute to this defect of personal nicety, of which I am now speaking, many or most of the peculiarities of habits and manners that have laid us open to foreign criticism—a criticism under whose taunts we wince with special and morbid sensitiveness.

In the advancing settlements of our new country, much may be pardoned to the condition and circumstances of the pioneer. But surely, under any contingencies, a Christian should wash his hands as often as a Musselman, or a Hindoo. Cool springs and running streams abound almost everywhere in our inhabited territory, whether of forest or prairie land, and our chief cities are supplied with fountains in royal munificence.

From neglect of these matters flows naturally a culpable-indifference to the neatness of clothing, the house, the table, and all other domestic arrangments. All these points of habit are consistent, and we can thus account for the nuisance of stained and slippery floors of the masticators of tobacco, which offend so many of our senses.

After showing the great increase of morality and consel quent shortening of the period of human life by the privations and moral pollutions of crowding human beings too closely together, by statistics of the most densely populated districts of Liverpool, London, and Boston, he shows the importance of legal enactments against such dense population, and especially that "all domiciliation in cellars be absolutely forbidden," observing that "these caves are unfit for the residence of domestic animals and fatal to man."

Altogether, it is a very instructing and highly interesting document.

A Plea for Obstetrics, is the title of Prof. Wiltbank's lecture. It is a clear and just statement of the importance of this branch of medicine. He shows it to be the most important to the practitioner on account of the great numbers of the population interested—all the women and children. Not only so, but future generations are to be born by the laws upon which it is founded, deviations from which it is designed to correct, and because the knowledge required is liable to be called into requisition in any case, having no time for preparation.

He shows too that the cultivation of obstetrics as a science, though it is younger than any other branch of our profession, has rewards equal to other departments for its votaries. He instances the eminent men of our own country, and shows correctly that amongst them all, there is no one who has acquired such a world-wide reputation as Dr. Dewees, a eulogium upon whose character closes the lecture.

Prof. Merrick's lecture is an exhordium to application in search of truth, with many good practical directions and pleasing encouragements. It is in fluent, chaste, and pleasing style, well calculated to gratify and profit his class.

Dr. Cooper's address shows energy of character in the author; and the plan for pursuing anatomical studies he has marked out, by forming a private class, is a good one. It would be well for the profession if private preceptors generally devoted more time to the instruction of students, as it would improve both. The Doctor is a little mistaken in reference to his character as a pioneer in the work of giving private courses, with dissections. Classes were assembled for such purposes in different places in the west, within our knowledge several years ago.

In setting forth the advantages offered by his course, Dr. Cooper says the dissections will continue as long as the weather will permit, which will enable them to acquire a proficiency seldom met with, and "during the course you will have an opportunity of witnessing and ass'sting in the performance of most of the important surgical operations on the dead subject, which will not only give you a delight in surgery, taking it as science, but it will overcome any natural repugnance which you may have to cutting human flesh, which is indispensable to your success as operative surgeons."

We have received Dr. Yandell's lecture, but unfortunately it has either been carried off or misplaced. E.

---

